# Adolescent Problem Gaming and Loot Box Purchasing in Video Games: Cross-sectional Observational Study Using Population-Based Cohort Data

**DOI:** 10.2196/23886

**Published:** 2021-02-09

**Authors:** Soichiro Ide, Miharu Nakanishi, Syudo Yamasaki, Kazutaka Ikeda, Shuntaro Ando, Mariko Hiraiwa-Hasegawa, Kiyoto Kasai, Atsushi Nishida

**Affiliations:** 1 Addictive Substance Project Tokyo Metropolitan Institute of Medical Science Tokyo Japan; 2 Research Center for Social Science & Medicine Tokyo Metropolitan Institute of Medical Science Tokyo Japan; 3 Graduate School of Medicine University of Tokyo Tokyo Japan; 4 School of Advanced Science SOKENDAI (Graduate University for Advanced Studies) Kanagawa Japan

**Keywords:** loot box purchasing, gambling, adolescents, primary caregivers

## Abstract

**Background:**

Video game loot boxes, which can typically be purchased by players or are given as reward, contain random virtual items, or loot, ranging from simple customization options for a player's avatar or character, to game-changing equipment such as weapons and armor. Loot boxes have drawn concern, as purchasing loot boxes might lead to the development of problematic gambling for adolescents. Although parental problem gambling is associated with adolescent problem gambling, no studies have evaluated the prevalence of loot box purchases in adolescents’ parents.

**Objective:**

This study investigated the association between loot box purchasing among adolescents and parents, and problem online gaming in population-based samples.

**Methods:**

In total, 1615 adolescent (aged 14 years) gamers from Japan responded to a questionnaire regarding their loot box purchasing and problem online gaming behaviors. Problem online gaming was defined as four or more of the nine addictive behaviors from the Diagnostic and Statistical Manual of Mental Disorders. The adolescents’ primary caregivers were asked about their loot box purchasing.

**Results:**

Of the 1615 participants, 57 (3.5%) reported loot box purchasing. This prevalence did not differ according to primary caregivers’ loot box purchasing, but adolescents who purchased loot boxes were significantly more likely to exhibit problem online gaming (odds ratio 3.75, 95% CI 2.17-6.48).

**Conclusions:**

Adolescent loot box purchasing is linked to problem online gaming, but not with parents’ loot box purchasing. Measures to reduce these behaviors should target reducing addictive symptoms in young video gamers.

## Introduction

Video game loot boxes have drawn concern over similarities to problem gambling [1]. A gambling disorder is typically characterized by adult issues with gambling. However, loot boxes feature heavily in video games marketed to children who pay for game items/rewards with real-world currency [2]. Loot boxes contain randomized content, and therefore their value is unclear at the time of purchase. Young people are more likely to exhibit impulsive behaviors and to find risk-taking appealing. Therefore, purchasing loot boxes might lead to developing problematic gambling among adolescents [3]. The relationship between problem gambling and loot box purchasing appears to be moderated by problem online gaming or excessive gaming [4,5].

The prevalence of loot box purchasing is poorly understood in adolescence. Most studies on the role of loot box purchasing in problem gambling used adult samples [4,6,7], with only two empirical studies utilizing adolescent samples. One study recruited participants via an online bulletin board, which may have included more varied gamers engaging in loot box activities instead of only adults [8]. Another study used a community sample from the Danish Civil Registration System [9]. However, no studies have evaluated the prevalence of loot box purchases in adolescents’ parents, even though parental problem gambling is associated with adolescent problem gambling [10-12]. It is unclear whether loot box purchases among adolescents are triggered by parental behavior or by their own problem online gaming. Such understanding constitutes an important basis for developing policies and interventions to prevent or mitigate the risks related to adolescent loot box purchasing.

This study investigated the association between loot box purchasing in adolescents and parents, and problem online gaming in population-based samples.

## Methods

### Participants

Data were obtained from the Tokyo Teen Cohort (TTC) study, an ongoing, prospective, and population-based birth cohort study on adolescents and their primary caregivers [13]. The TTC investigates adolescents’ health and development, with the details of the study described elsewhere [14-16]. All procedures involving human participants were performed in accordance with the ethical standards of the associated institutional research committees, and in adherence to the 1964 Helsinki Declaration and its later amendments or comparable ethical standards. Informed consent was obtained from all adolescent respondents and their caregivers included in the study. The TTC’s study protocol was approved by the institutional review boards of the Tokyo Metropolitan Institute of Medical Science (approval number 12–35), SOKENDAI (Graduate University for Advanced Studies; 2012002), and the University of Tokyo (10057).

This study sample included 2667 adolescents (aged 14 years) born between September 2002 and August 2004, along with their primary caregivers. Data were collected via a self-reported questionnaire.

### Procedures

In the questionnaire, adolescents and their primary caregivers were asked whether they played online video games. Participants who answered “yes” were defined as “gamers,” who then answered further questions regarding loot box purchasing. A single question was used to determine whether they spent real money on items/rewards in games, excluding payment for game consoles and software fees.

**Figure 1:**
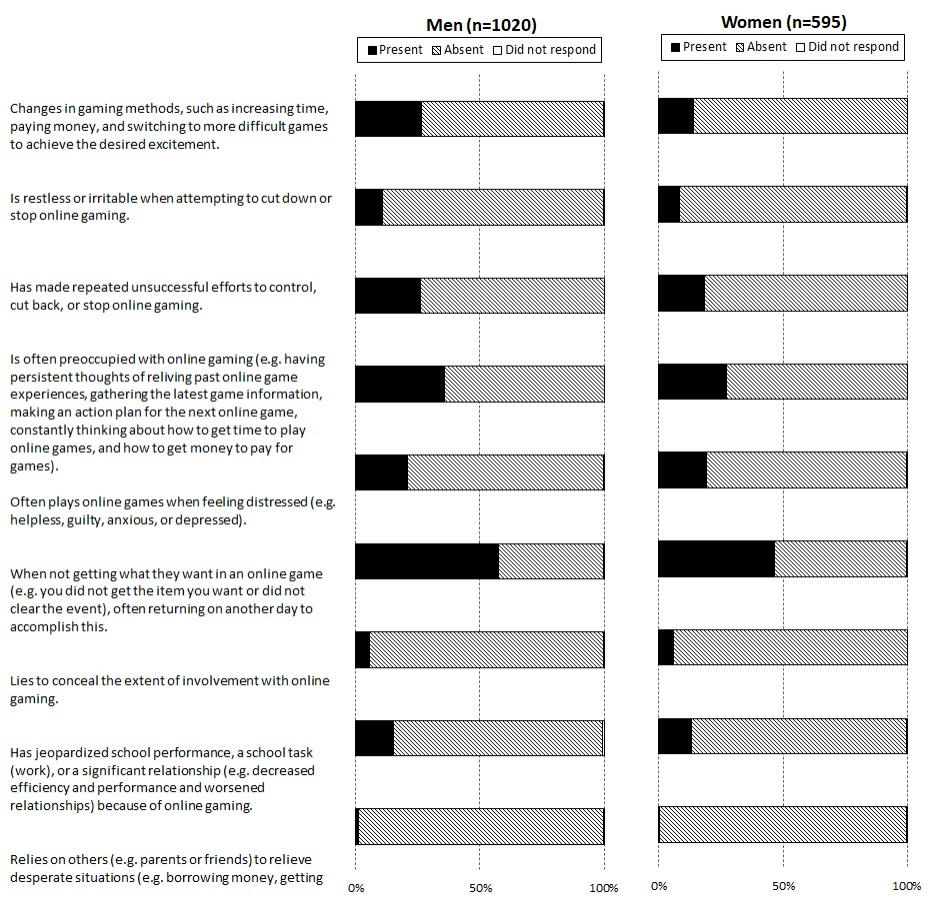
Presence of problem gambling behaviors among adolescent gamers.

Adolescents were also asked about problem online gaming. The proposed criteria for gambling disorder in the Diagnostic and Statistical Manual of Mental Disorders, Fifth Edition were used to assess problem online gaming. The criteria were restated as nine questions (Figure 1) to assess the presence of certain behaviors or emotions related to addiction in online games within the past 12 months. Based on the proposed criteria, respondents who reported four or more behaviors were defined as having problem online gaming.

### Statistical Analysis

Odds ratios (ORs) of problem online gaming according to the presence of loot box purchasing were calculated using univariate binomial logistic analysis. As the association between loot box engagement and problem online gaming is stronger for women than men [8,9], the analysis was stratified by sex.

## Results

**Figure 2:**
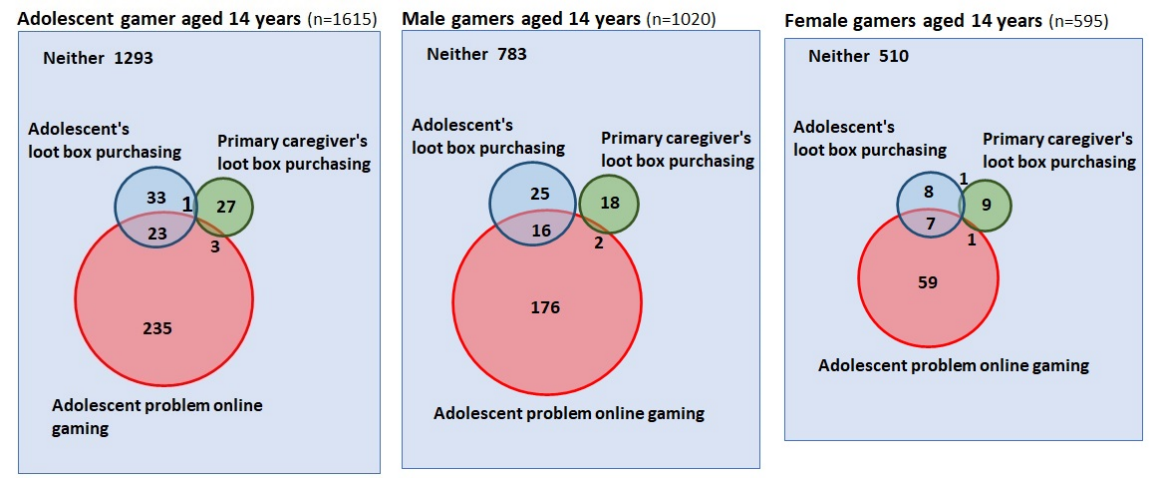
Venn diagrams of loot box purchasing and problem gambling in adolescent gamers.

Of the 2619 respondents (1392 men, 1227 women), 1615 adolescents answered that they played online games (1020 men, 595 women). Of these 1615 gamers, 57 (3.5%) reported purchasing loot boxes (4.0%, 41/1020 men; 2.7%, 16/595 women). Of the 1615 primary caregivers of adolescent gamers, 514 (31.8%) played online games and 31 (6.0%) reported purchasing loot boxes. The prevalence of adolescent loot box purchasing did not vary according to primary caregivers’ loot box purchasing (*χ*^2^_1_=0.009, *P*=.93; Figure 2).

Of the 1615 gamers, 261 (16.2%) exhibited four or more addictive symptoms in online gaming (194/1020, 19.0% men; 67/595, 11.3% women); 23 adolescents reported purchasing loot boxes and met the criteria for problem online gaming (Figure 2). Logistic regression analysis revealed that the likelihood of problem online gaming was significantly higher for adolescent gamers who purchased loot boxes than for those who did not (OR 3.75, 95% CI 2.17-6.48). The OR was greater in female gamers (OR 6.73, 95% CI 2.42-18.72) than in male gamers (OR 2.88, 95% CI 1.51-5.51).

## Discussion

### Principal Findings

Loot box purchasing was reported by 3.5% of 1615 gamers aged 14 years, whereas only 1.9% of their parents reported this behavior. Parental loot box purchasing did not correlate with that of adolescents. Problem online gaming was more frequently observed, which was evident in 15.7% of adolescent gamers, and those who purchased loot boxes were significantly more likely to exhibit problem online gaming.

The link between loot box purchasing and problem online gaming found in this study is consistent with previous reports in adult populations [4,5]. As reported in previous studies [8,9], male adolescents were more likely to be gamers and purchase loot boxes than female gamers. However, unlike other gambling behaviors, adolescents’ loot box purchasing does not appear to be inherited from their parents. Measures to reduce loot box purchases should target reducing addictive symptoms in young online gamers.

Our study’s strength lies in confirming the absence of a correlation between the loot box purchasing behaviors of early adolescents and those of their parents. Multifaceted support for self-regulation in combination with parenting techniques for limiting loot box purchases is recommended for children gamers [17]. Our findings suggest that parental techniques to prevent problem online gaming may also be helpful to reduce loot box purchases.

### Limitations

This study has some limitations. The cross-sectional design did not allow us to draw conclusions regarding the causal relationship between loot box purchasing and problem online gaming. A longitudinal study would be beneficial to evaluate how loot box purchasing in early adolescence correlates with other addictive behaviors in later adolescence and with gambling in adulthood.

### Conclusions

Parental loot box purchasing did not correlate with that of adolescents. Adolescents who purchased loot boxes were significantly more likely to exhibit problem online gaming. Measures to reduce loot box purchases should target reducing addictive symptoms in young video gamers.

## Abbreviations

OR: odds ratio
TTC: Tokyo Teen Cohort
